# PdWND3A, a wood-associated NAC domain-containing protein, affects lignin biosynthesis and composition in *Populus*

**DOI:** 10.1186/s12870-019-2111-5

**Published:** 2019-11-11

**Authors:** Yongil Yang, Chang Geun Yoo, William Rottmann, Kimberly A. Winkeler, Cassandra M. Collins, Lee E. Gunter, Sara S. Jawdy, Xiaohan Yang, Yunqiao Pu, Arthur J. Ragauskas, Gerald A. Tuskan, Jin-Gui Chen

**Affiliations:** 1 BioEnergy Science Center and Biosciences Division, Oak Ridge National Laboratory, Oak Ridge, TN 37831 USA; 2 Center for Bioenergy Innovation, Oak Ridge National Laboratory, Oak Ridge, TN 37831 USA; 3 UT-ORNL Joint Institute for Biological Science, Oak Ridge National Laboratory, Oak Ridge, TN 37831 USA; 4 ArborGen Inc., Ridgeville, SC 29472 USA; 5 Department of Chemical and Biomolecular Engineering & Department of Forestry, Wildlife, and Fisheries, University of Tennessee, Knoxville, TN 37996 USA

**Keywords:** F5H, Lignin, *Populus*, Saccharification, Sinapyl alcohol, S/G ratio, VND

## Abstract

**Background:**

Plant secondary cell wall is a renewable feedstock for biofuels and biomaterials production. *Arabidopsis* VASCULAR-RELATED NAC DOMAIN (VND) has been demonstrated to be a key transcription factor regulating secondary cell wall biosynthesis. However, less is known about its role in the woody species.

**Results:**

Here we report the functional characterization of *Populus deltoides* WOOD-ASSOCIATED NAC DOMAIN protein 3 (PdWND3A), a sequence homolog of *Arabidopsis* VND4 and VND5 that are members of transcription factor networks regulating secondary cell wall biosynthesis. *PdWND3A* was expressed at higher level in the xylem than in other tissues. The stem tissues of transgenic *P. deltoides* overexpressing *PdWND3A* (*OXPdWND3A*) contained more vessel cells than that of wild-type plants. Furthermore, lignin content and lignin monomer syringyl and guaiacyl (S/G) ratio were higher in *OXPdWND3A* transgenic plants than in wild-type plants. Consistent with these observations, the expression of *FERULATE 5-HYDROXYLASE1 (F5H1)*, encoding an enzyme involved in the biosynthesis of sinapyl alcohol (S unit monolignol), was elevated in *OXPdWND3A* transgenic plants. Saccharification analysis indicated that the rate of sugar release was reduced in the transgenic plants. In addition, *OXPdWND3A* transgenic plants produced lower amounts of biomass than wild-type plants.

**Conclusions:**

PdWND3A affects lignin biosynthesis and composition and negatively impacts sugar release and biomass production.

## Background

Plant cell walls define cellular space and protect internal cellular component against extracellular biotic and abiotic stimuli. In addition to the structural roles, plant cell walls have become an attractive target for conversion into biofuels and biomaterials due to their abundance, alternate chemical composition properties, and renewability. Plant cell walls are generally composed of two types of walls, i.e., the primary cell wall and secondary cell wall. The primary cell wall typically consists of cellulose, hemicellulose and pectin whereas the secondary cell wall contains a larger proportion of lignin [22, 56]. Of these secondary cell wall components, cellulose and hemicellulose are polysaccharides and are being considered as substrates for conversion into biofuels [4, 10, 26]. Lignin as a polyphenolic biopolymer contributes to cell rigidity and protection against pathogens [3]. In addition, lignin facilitates hydrophilic transport by coating the interior of vessels which helps regulate water relations in the plant. However, from the perspective of biofuels production lignin is regarded as a major recalcitrance factor limiting access to cell wall polysaccharides. Therefore, genetic modification of lignin biosynthesis pathway has become an effective approach for reducing recalcitrance and improving biofuel conversion and production.

Phenylpropanoids, derived from phenylalanine, are the pivotal metabolic precursors to monolignol synthesize [16, 29, 45]. The general phenylpropanoid pathway includes three initial steps that are catalyzed by L-phenylalanine ammonia-lyase (PAL), cinnamate 4-hydroxylase (C4H) and 4-coumarate:CoA ligase (4CL) [15, 35, 37]. 4-coumaryl-CoA is the final product of general phenylpropanoid pathway and is the precursor chemical for synthesizing three different chemical families, i.e., flavonoids, monolignols, and phenolic acids. The lignin biosynthetic pathway has been well characterized and most biosynthetic enzymes have been identified [2, 29, 44]. Lignin is composed of three monomers known as hydroxyphenyl (H), guaiacyl (G) and syringyl (S) that are derived from *p*-coumaryl, coniferyl and sinapyl alcohols, respectively, and whose productions are regulated by caffeoyl-CoA O-methyltransferase (CCoAOMT), ferulate 5-hydroxylase (F5H), cinnamoyl CoA reductase (CCR), and cinnamoyl alcohol dehydrogenase (CAD) [12, 14, 25, 29, 31, 52]. The regulation and expression of lignin biosynthetic genes is associated with several transcription factors, including NAC (No Apical Meristem (NAM), *Arabidopsis* Transcriptional Activation Factor (ATAF1/2), Cup-shaped Cotyledon (CUC2)), and V-myb myeloblastosis viral oncogene homolog (MYB) [11, 16, 56]. Of these transcription factors, NAC family proteins function as the master switch regulator of secondary cell wall formation. Kubo et al. [13] suggested that the NAC transcription factors of VASCULAR-RELATED NAC-DOMAIN (VND) 1–7 subfamily act as master regulators of meta and proto xylem vessel formation in *Arabidopsis* root. NAC SECONDARY WALL THICKENING PROMOTING FACTOR1 (NST1) and NST3/SECONDARY WALL-ASSOCIATED NAC DOMANIN PROTEIN1 (SND1) have also been shown to act as master transcriptional regulators of secondary cell wall formation and fiber cell differentiation [13, 20, 21, 23, 41, 47]. SND1 has been reported to bind directly to the promoter of *MYB46* [53]. SND1 also acts as a switch to regulate the expression of many downstream genes related to the secondary cell wall biosynthesis including cellulose and lignin biosynthesis. In *Arabidopsis*, the intricate network of transcriptional regulation of secondary cell wall biosynthesis has been summarized in several recent review articles [16, 22, 49, 55, 56].

As NAC family members, *Arabidopsis* VND 1–7 (AtVND1–7) were initially identified in the early stage of xylem vessel cell trans-differentiation using *Arabidopsis* suspension cultures [5]. The transgenic *Arabidopsis* overexpressing *AtVND1–7* resulted in ectopic formation of xylem vessel element [6, 13, 39, 58]. The comparative transcriptome analysis of inducible expression of AtVND6 and AtSND1 in transgenic *Arabidopsis* system showed that the upregulated genes by AtVND6 were overlapped with those genes by AtSND1 [23]. However, there were also genes that were preferentially regulated by AtVND6 or AtSND1 [23]. Furthermore, a total of 63 genes encoding a broad range of proteins, including both transcription factors and non-transcription factors involved in the programmed cell death were identified as target genes of AtVND7 in an overexpression study [40]. Therefore, AtVNDs share with AtSND a common set of downstream target genes but also regulate the expression of target genes that are distinct from those regulated by AtSND. Electrophoretic mobility shift assay of AtVND1–7 and transactivation analysis of AtVND6 and AtVND7 showed that AtVNDs bind to the 19-bp consensus DNA sequence of secondary wall NAC binding element (SNBE) and the 11-bp tracheary-element-regulating *cis*-elements (TERE) in the promoter region of a group of genes involved in the secondary cell wall biosynthesis, cell wall modification, and programmed cell death [6, 23, 48]. Both TERE and SNBE were also found in the promoter sequences of some direct target genes of AtSND1 [23, 28, 48, 50, 51].

In the woody perennial species *Populus trichocarpa*, a total of eight genes among 16 *Populus* NAC domain protein genes were sub-grouped as *Populus* VND (PtrWND/PtVNS) [22, 24, 48]. Dominant repression of PtrWND2B/PtVNS10 and PtrWND6B/PtVNS08 using EAR-induced dominant repression approach in hybrid *Populus* (*P. tremula* × *P. alba*) resulted in reduction of wall thickness of xylary fibers [51], whereas ectopic secondary wall thickening phenotype was observed in transgenic *Populus* plants overexpressing all *PtrWND*/*PtVNS* genes driven by cauliflower mosaic virus 35S promoter [24]. Moreover, ectopic deposition of lignin, cellulose, and hemicellulose was observed in transgenic *Arabidopsis* and *Populus* overexpressing *PtrWND6B* (an *AtVND7* homolog) [48, 51]. Therefore, *Populus* VND-related proteins (PtVND) appeared to function similarly as AtVND in the regulation of vascular vessel formation and secondary cell wall biosynthesis [24, 48, 51]. This was further supported by the observation that heterologous expression of PtrWND3A/PtVNS05 and PtrWND3B/PtVNS06 (AtVND4 and 5 homologs) in *Arabidopsis* resulted in ectopic secondary wall deposition in leaf [24].

Here we report the functional characterization of PdWND3A, an AtVND4/5 sequence homolog, using *Populus* transgenics. The transgenic *Populus* overexpressing *PdWND3A* displayed increased vessel formation in the stem. Both lignin content and lignin S/G ratio were increased in the transgenic plants. Interestingly, RT-PCR analysis indicated that among tested secondary cell wall biosynthesis-related genes, the expression of *F5H1* was predominantly up-regulated in the transgenic plants, suggesting that PdWND3A may affect lignin biosynthesis and composition by regulating *F5H1* expression.

## Results

### Phylogenetic analysis of *Populus* NAC domain-containing proteins

In a previous studies, Zhong and Ye [54] used AtSND1 (AT1G32770) as a template to search for *Populus* homologs and defined their nomenclatures. In another study, Ohtani et al. identified 16 NAC domain protein genes in the *Populus* genome based on the protein homology analysis with *Arabidopsis* VND/NST/SND protein [24]. With the availability of the latest *P. trichocarpa* genome annotation (v3.1), we used AtSND1 as a template to search of all possible AtSND1 sequence homologs in Phytozome (https://phytozome.jgi.doe.gov) [8] and identified a total of 21 *Populus* loci with a cutoff of amino acid sequence identity > 30% (Additional file 2). Among these proteins, a group of eight *Populus* proteins showing high amino acid sequence identity with respective AtVND proteins were selected for further study (Additional file 3). Two clades, including four *Populus* proteins (Potri.012G126500, Potri.015G127400, Potri.001G120000 and Potri.003G113000), shared a cluster with AtVND4 and AtVND5. On the basis of these results, we selected Potri.015G127400, which was previously designated as PtrWND3A [54] for further characterization. PtrWND3B (Potri.012G126500), in the same clade with PtrWND3A, shared 95.3% similarity with PtrWND3A at the amino acid level (Additional file 3). A DNA fragment of 24 bp is absent in the middle of coding sequence of *PtrWND3A*; therefore, we were able to use gene-specific primer for this region to distinguish *PtrWND3A* from *PtrWND3B* (Additional file 4). Similar to *Arabidopsis* VND proteins, NAC domain at the N-terminus of PtrWND3A is the only predictable domain (Additional file 3).

### Expression pattern of *PdWND3A*

**Fig. 1:**
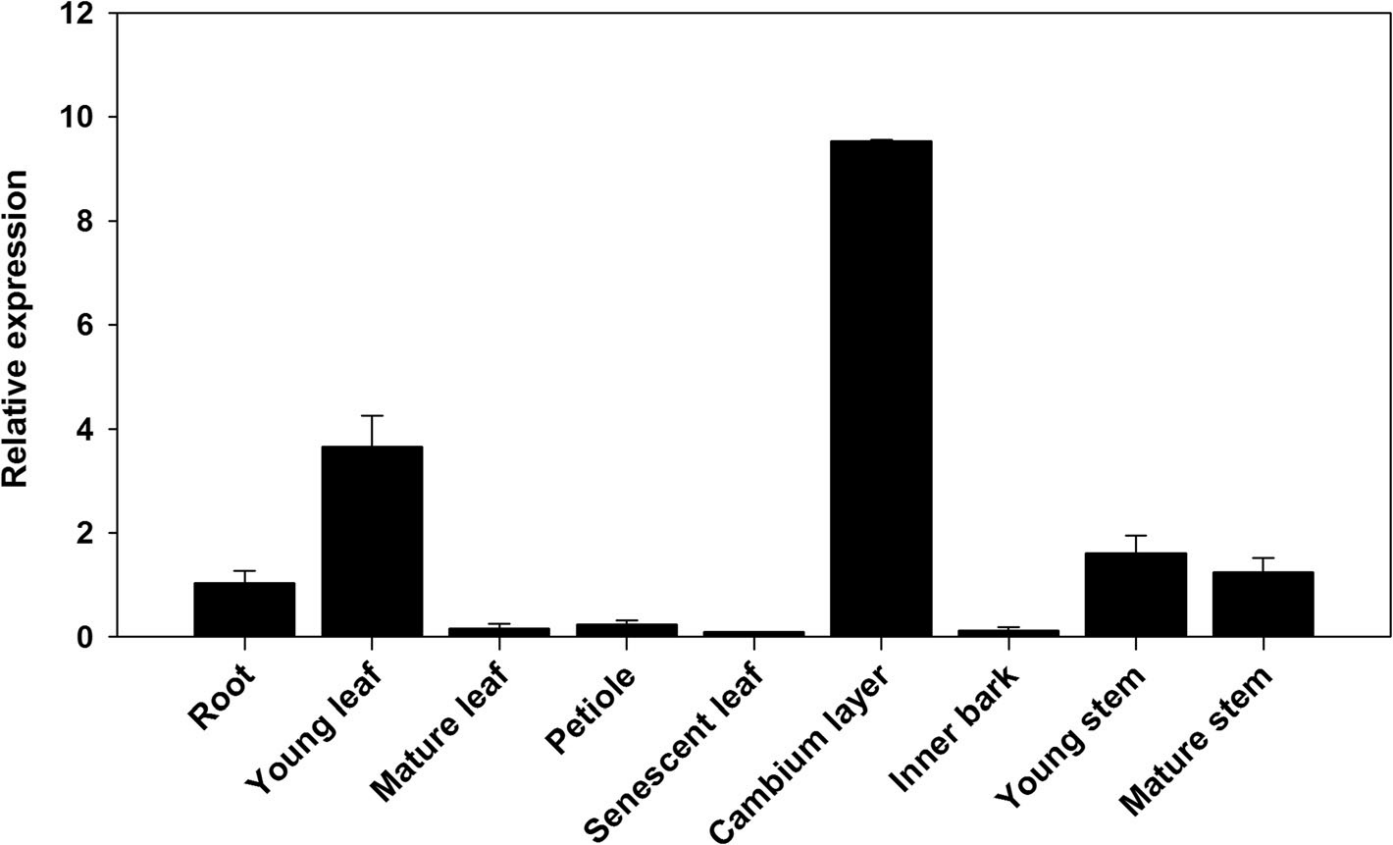
Expression of *PdWND3A* in various tissues and organs. Different tissues and organs were collected between 12:00 PM and 2:00 PM from three WV94 plants. Total RNAs were extracted from root, young leaf, mature leaf, young stem (internodes 1 to 3), mature stem (internodes 6 to 8), petiole of mature leaf, inner bark (bark of mature stem) and cambium layer (scrapped stem under bark of mature stem). The *PdWND3A* transcript level was measured by qRT-PCR. Shown are mean values of three biological replicates ± S.D.

To functionally characterize *PdWND3A*, we first examined *PdWND3A* transcript abundance in various tissues and organs by using quantitative RT-PCR. *PdWND3A* transcript was detected in all tested tissues and organs with the highest abundance in the xylem, suggestive of a role in secondary cell wall biosynthesis (Fig. 1).

### Transgenic *Populus* plants overexpressing *PdWND3A*

**Fig. 2:**
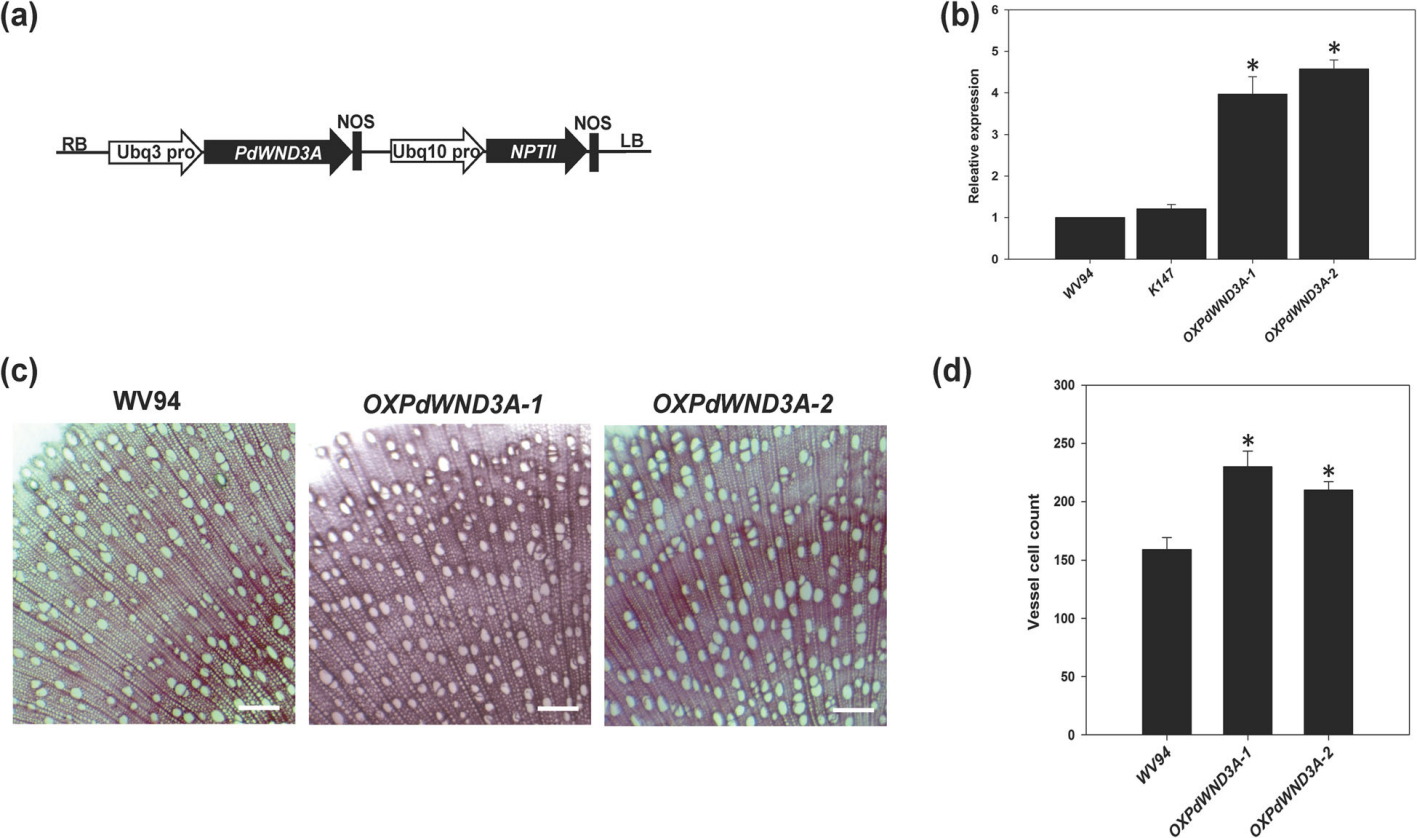
*Populus* transgenic plants overexpressing *PdWND3A* (*OXPdWND3A*). **a** The gene construct used to generate transgenic plants in *P. deltoides* WV94 background overexpressing *PdWND3A*. **b** Quantitative RT-PCR analysis of *PdWND3A* transcript level in the transgenic lines. Leaf tissues were used for RNA extraction. **c** Phloroglucinol-HCl staining image of stem cross-section of 6-month-old *OXPdWND3A* transgenic plants. **d** Vessel number. The vessel number was counted in 1 cm^2^ area in the microscopic image. Shown are the mean values of three biological replicates ± S.D. The asterisk marks the statistical significance against WV94 (*p* < 0.01, *n* = 3)

Subsequently, we generated transgenic *P. deltoides* plants overexpressing *PdWND3A* (Fig. 2a). A total of 14 independent transgenic lines were generated and six of them were confirmed to overexpress *PdWND3A* (Additional file 5). We selected two independent lines for further analyses. These two transgenic lines were designated as *OXPdWND3A-1* and *OXPdWND3A-2* and were confirmed by quantitative RT-PCR to be *PdWND3A* overexpressors (Fig. 2b).

AtVND family proteins are viewed as master switch transcription factors regulating vessel formation in xylem tissue [22]. To examine whether such a function is conserved in *Populus*, we examined vessel formation in *OXPdWND3A* transgenic plants. Cytological analysis with cross-section specimen of mature stem revealed a dense vessel formation in the stem of *OXPdWND3A* (Fig. 2c), with the number of xylem vessel significantly higher in *OXPdWND3A* transgenic plants compared to wild-type WV94 (Fig. 2d). These results support the view that the regulation of vessel formation is a common function of VND proteins in both *Arabidopsis* and *Populus*.

### Chemical composition analysis of secondary cell wall components in *OXPdWND3A* transgenic plants

**Fig. 3:**
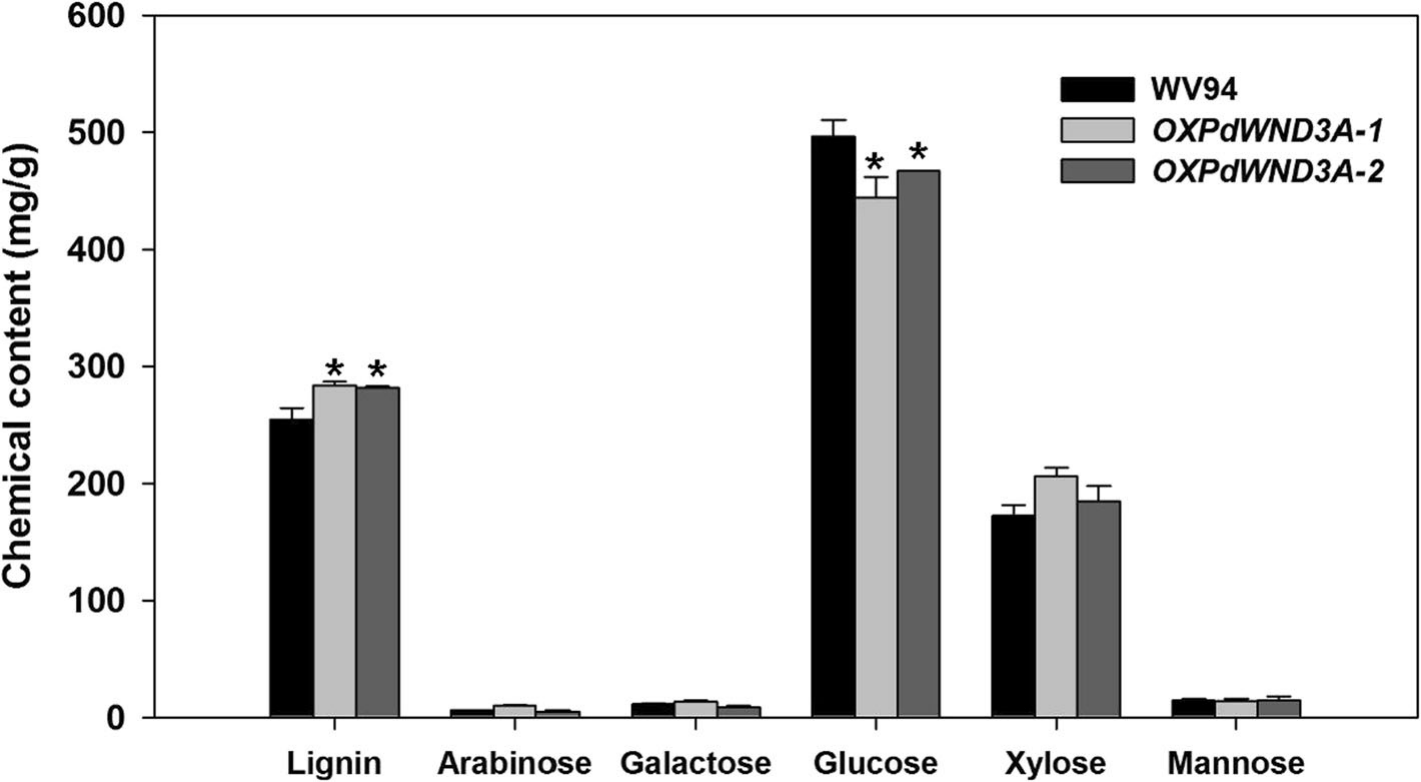
Chemical composition in the stem of 6-month-old *OXPdWND3A* transgenic plants**.**The de-barked stem of 6-month-old *OXPdWND3A* transgenic plants was Wiley-milled and subjected to chemical composition analysis using ion chromatography. Shown are the mean values of three biological replicates ± S.D. The asterisk marks the statistical significance against WV94 (*p* < 0.01, *n* = 3)

To examine possible changes in the contents of secondary cell wall components in the stem tissue of *OXPdWND3A*, we performed chemical composition analysis. We found that *OXPdWND3A* lines had significantly higher lignin content than WV94 (*p* < 0.01; Fig. 3). As a predictable monosaccharide from cellulose, the glucose content was reduced in *OXPdWND3A* lines while no significant difference was observed in xylose content (Fig. 3). The contents of other chemical components such as arabinose and mannose were also not altered in *OXPdWND3A*s compared to WV94 (Fig. 3).

### Lignin physicochemical characterization

**Fig. 4:**
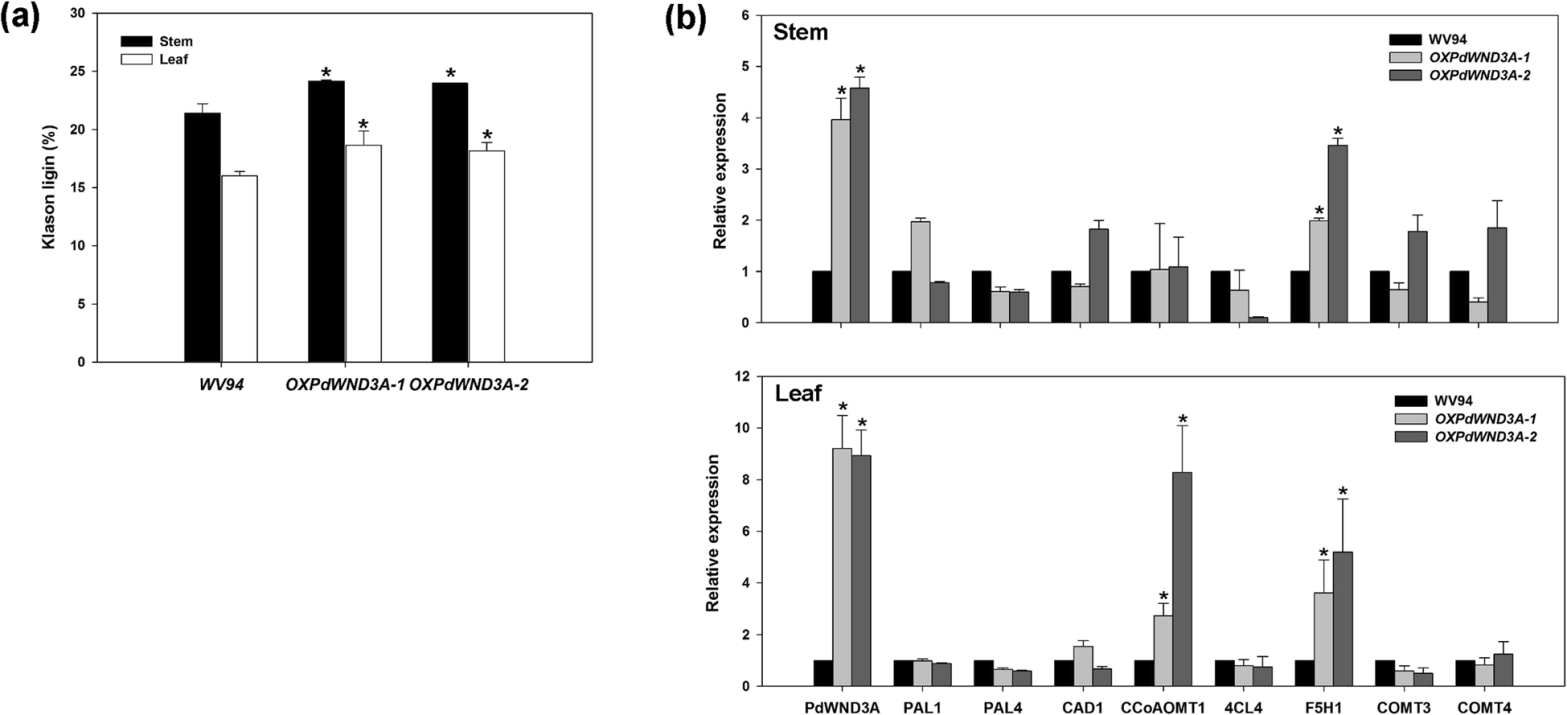
Analyses of Klason lignin content and the expression of lignin biosynthesis-related genes in *OXPdWND3A* transgenic plants. **a** Klason lignin (acid-insoluble lignin) content detected from leaf and stem tissues. **b** Relative gene expression of representative lignin biosynthesis-related genes in *OXPdWND3A* transgenic plants. Shown are the mean values of three biological replicates ± S.D. The asterisk marks the statistical significance against WV94 (*p* < 0.01, *n* = 3)

Because lignin content was increased in the *OXPdWND3A* transgenic plants (Fig. 3), we sought further evidence supporting a role of *PdWND3A* in lignin biosynthesis. We performed Klason lignin (acid insoluble lignin) analysis with both leaf and stem tissue of the same plants. The Klason lignin content in stem tissue of *OXPdWND3A*-*1 and OxPdWND3A-2* were 12.71 and 11.89% higher than wild-type WV94, respectively (Fig. 4a). *OXPdWND3A-1* and *OXPdWND3A-2* also contained 16.42 and 13.36% more lignin in leaf tissue compared to WV94, respectively (Fig. 4a).

To further examine the structural characteristics of lignin, the 2D ^1^H-^13^C NMR analysis was conducted with stem tissue. The *OXPdWND3A* had relatively high S content and low G content compared to those of WV94, resulting in higher S/G ratio (Table 1). The chemical linkage between lignin subunit was altered in the stem of *OXPdWND3A*. The relative content of the carbon-carbon bond such as resinol (β-β) linkages was significantly increased in *OXPdWND3A* compared to WV94 (Table 1).

**Table 1:** Analysis of lignin monolignols and interunit linkage in *OXPdWND3A* transgenic plants. Structural information of lignin was obtained by 2D ^1^H-^13^C HSQC NMR analysis. The contents of monolignols (S and G), PB (*p*-hydroxybenzoate), and lignin interunit linkage [β-aryl ether (β-*O*-4), phenylcoumaran (β-5), and resinol (β-β)] were calculated as a fraction of total lignin subunits (S + G). Two biological replicates of stem tissues were used for the analysis. The number in parenthesis displayed standard deviation

	WV94 Control	*OXPdWND3A-1*	*OXPdWND3A-2*
Lignin subunits			
S	56 (0.81)	61 (3.89)^*^	67 (1.12)^*^
G	44 (0.81)	39 (2.89)^*^	33 (1.12)^*^
S/G	1.29	1.54	2.07
PB	7 (0.45)	10 (0.21)	2 (0.59)
Lignin interunit linkages			
β-*O*-4	54 (2.19)	58 (3.74)	59 (4.39)
β-5	2 (0.78)	4 (1.39)	2 (1.45)
β-β	2 (0.83)	4 (0.46)^*^	6 (0.5)^*^

^*^Statistical significance against WV94 (*p* < 0.05)

### Gene expression analysis

To determine whether PdWND3A impacts expression of genes involved in lignin biosynthesis or monomer composition, we performed quantification analysis of the expression of lignin biosynthetic genes. We measured the relative transcript abundance of eight representative lignin biosynthetic genes, including *PAL1* and *PAL4*, *CAD1* and *4CL* for general phenylprophenoid pathway, and *CCoAOMT1*, *F5H1, COMT3* and *COMT4* for monolignol biosynthesis. Among these eight tested genes, *F5H1*, a gene involved in S unit lignin monomer biosynthesis, was upregulated in both leaf and young stem tissues in the transgenic lines compared to the wild-type control (Fig. 4b). Although COMT and CAD had been reported to regulate S unit lignin monomer biosynthesis [27], no significant difference in their transcript level was observed between *OXPdWND3A* transgenic plants and the wild-type WV94. The transcript level of *CCoAOMT1*, another key enzyme involved in the monolignol biosynthesis of G- and S-type lignin [18, 19], in leaf tissue was also higher in the transgenic plants than WV94. Other tested genes were not altered in either tissues between the transgenic lines and WV94 (Fig. 4b). Collectively, these results support that PdWND3A has a role in regulating the expression of genes involved in lignin biosynthesis and lignin monomer composition.

### Saccharification efficiency of *OXPdWND3A*

**Fig. 5:**
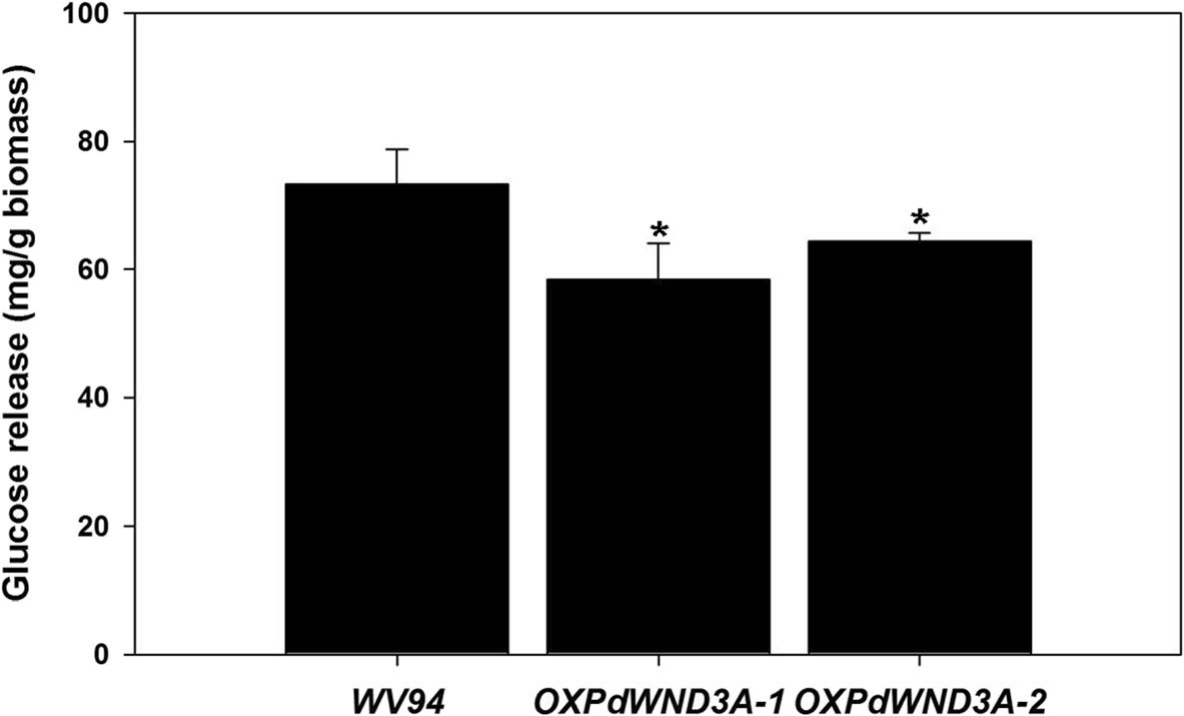
Saccharification efficiency of *OXPdWND3A* transgenic plants**.** Debarked and dried *Populus* stem was treated with cellulase enzyme for 48 h. The rate of glucose release was calculated from the detected data by the ion chromatography system. Shown are the averages of two biological replicates ± S.D.

The saccharification efficiency is an important indicator of the usefulness of genetically modified plant biomass for biofuel production. It is generally recognized that high lignin content negatively impacts saccharification efficiency [34]. In contrast, in hardwood species, higher S-to-G (S/G) ratio is often considered to be a factor positively influencing saccharification efficiency [34, 43]. *OXPdWND3A* transgenic lines have higher lignin content and higher S/G ratio compared to the control. Therefore, we wanted to examine how these two contrasting factors (i.e., high lignin content and high S/G ratio) impact saccharification efficiency. We measured glucose release, without pretreatment, by enzyme treatment for 48 h. The *OXPdWND3A* transgenic plants displayed lower glucose release compared to the control (Fig. 5). Therefore, in *OXPdWND3A* transgenic lines high lignin content appeared to dominate over high S/G ratio in the process of saccharification.

### *OXPdWND3A* biomass production

**Fig. 6:**
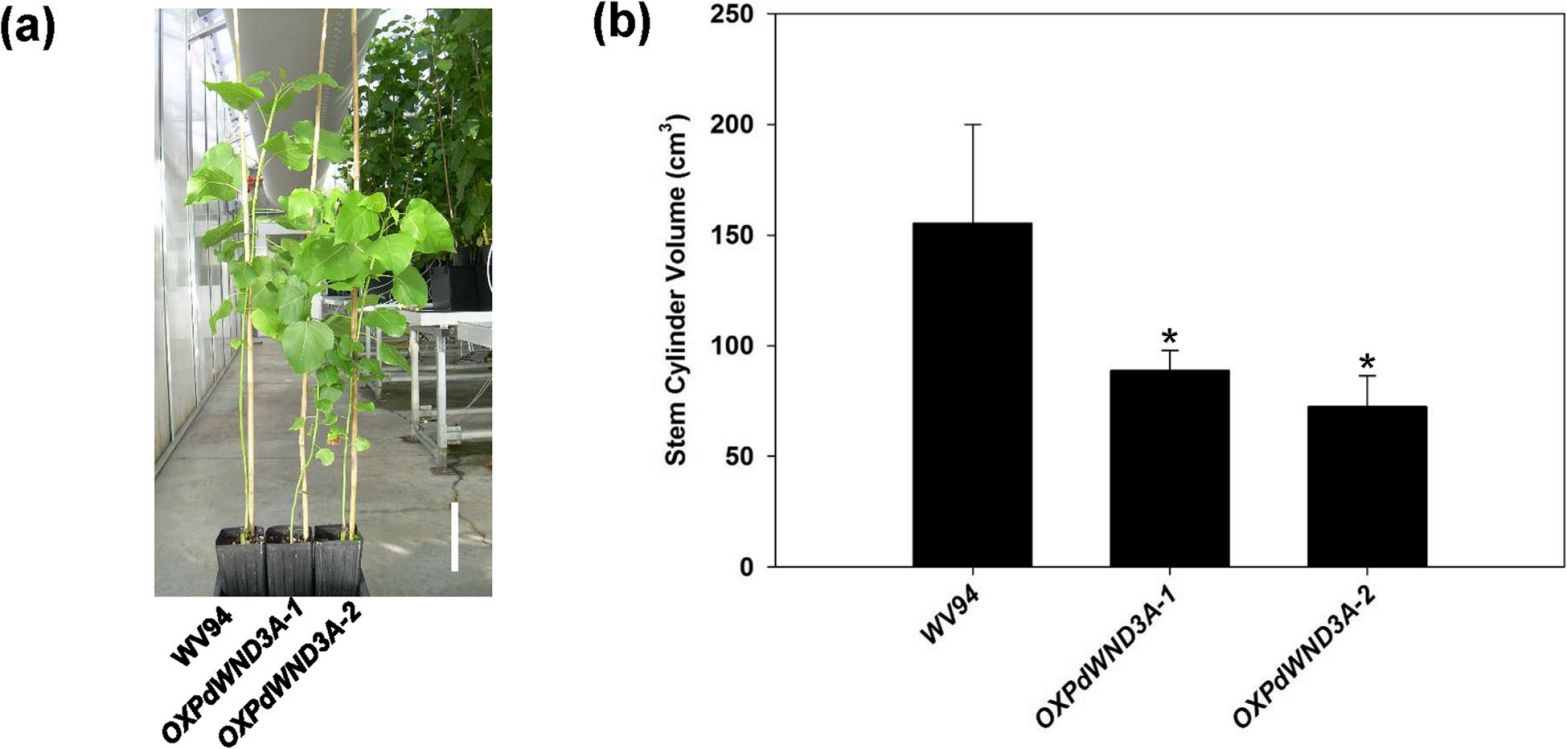
Biomass production of *OXPdWND3A* transgenic plants. **a** The whole plant picture of 3-month-old plants grown under greenhouse conditions. Scale bar, 25 cm. **b** The stem volume of *OXPdWND3A* transgenic plants compared to WV94. Shown are the average of estimated stem volume ± S.D. (*n* = 5) by using the πr^2^h equation with height and diameter of primary stem. The asterisk marks the statistical significance against WV94 (*p* < 0.01, *n* = 5)

Finally, to determine whether overexpression of *PdWND3A* affects biomass production, we measured the diameter and height to estimate stem volume. The overall plant stature of *OXPdWND3A* was smaller than wild-type WV94 plants grown under greenhouse conditions (Fig. 6a). The stem volume of both transgenic lines was significantly smaller than WV94 (Fig. 6b), suggesting that overexpression of *PdWND3A* negatively affects biomass production.

## Discussion

Woody biomass is mainly composed of cellulose, hemicellulose and lignin. The development of applicable method to convert biomasses to biofuel has been regarded as a pivotal research for cost-effective biofuel production. In the last decade, molecular and genetic studies of woody plants suggested that transcription factors are critical for regulating secondary cell wall biosynthesis. Of these transcription factors, NAC family proteins are viewed as master switches [22, 56]. We provide evidence here that PdWND3A, a member of NAC domain-containing protein family, is involved in the regulation of lignin biosynthesis and composition.

### PdWND3A and lignin biosynthesis and composition

The physicochemical analysis of *OXPdWND3A* transgenic lines suggest that overexpression of *PdWND3A* affects lignin biosynthesis (Fig. 4). Consistent with the physicochemical analysis, the histochemical image showed more xylem vessel formation in *OXPdWND3A* than WV94 (Fig. 2c and d). In earlier reports, overexpression of PtrWND6B, a homolog of AtVND7 protein, induced ectopic deposition of lignin in leaf epidermal and mesophyll cells [48, 51]. In addition, inducible expression of AtVND6 or AtVND7 in *Populus* resulted in ectopic lignin deposition [39]. More specifically, overexpression of *PtrWND3A* was shown to induce ectopic secondary cell wall deposition in poplar leaves [24]. Although a microscopic examination of ectopic secondary cell wall deposition in the leaves of transgenic plants overexpressing *PdWND3A* was not conducted in the present study, we provided physicochemical analysis which confirms that the lignin is accumulated in both the leaf and the stem of *OXPdWND3A* transgenic plants (Fig. 4a). In addition, monolignol composition between S and G unit was altered by overexpression of PdWND3A (Table 1). The structural properties in lignin were also altered in *OXPdWND3A*. We observed significant increase of resinol (β-β) linkages (Table 1). It was reported that β-β linages are primarily associated with S unit whereas phenylcoumaran is associated with G unit [33]. Therefore, the observed increased lignin resinol abundance is consistent with the observed increased lignin S/G ratio in *OXPdWND3A* transgenic lines. Collectively, these results suggest that PdWND3A is involved in the regulation of both lignin biosynthesis and lignin monomer composition. It remains unclear whether PdWND3A preferentially regulates lignin biosynthesis or composition and how PdWND3A achieves it. Because PdWND3A functions as a transcription factor, it may do so through the regulation of specific lignin pathway genes.

### PdWND3A and *F5H1* expression

Gene expression analysis of lignin biosynthetic genes in *OXPdWND3A* transgenic plants indicated that among all tested genes, *PdWND3A* overexpression primarily affected the expression of *F5H1* in both stem and leaf. This was in contrast to previous studies in *Arabidopsis* in which overexpression of every *AtVND* gene (*AtVND1* to *AtVND7*) was shown to induce the expression of *PAL1*, *CCoAOMT1* and *4CL* of lignin biosynthetic genes but not *F5H1* [23, 40, 58]. Zhou et al., [58] demonstated that the promoters of *CCoAOMT1* and *4CL* are directly activated by AtVND proteins (AtVND1 to AtVND5). In another study, *Populus* transgenic plants expressing *AtVND7* showed increased expression of genes encoding cationic peroxidase, laccase, CCR, and phenylcoumaran benzylic ether reductase related to lignin biosynthesis [24]. *Arabidopsis* transgenic plants expressing PtrWND6B, a *Populus* homolog of AtVND6, also showed increased expression of *4CL1* and *CCoAMT1* [54]. The transactivation assay using PtrWND6B as the effector construct identified laccase, *CCoAMT1* and *COMT1* as direct target genes of PtrWND6B [24, 48]. Therefore, the regulation of gene expression of lignin biosynthetic genes by VND homologs appeared to be conserved between *Arabidopsis* and *Populus*. However, no report had shown the relationship between *F5H1* expression and VND in previous studies. Our study showed that the expression of *F5H1* is upregulated by PdWND3A, implying that there may be regulatory specificity among members of VND/WND transcription factor family regarding their downstream direct or indirect target genes.

F5H mediates the chemical conversion from coniferaldehyde to 5-OH coniferaldehyde in the S monolignol biosynthesis pathway [33]. Overexpression of F5H from *Liquidambar styraciflua* in *Pinus radiate* produced more sinapyl alcohol in the lignin polymer [36]. Accumulative evidence suggested that the regulation of *F5H1* gene expression may be distinct from the common regulation of other lignin biosynthetic genes. For example, overexpression of AtMYB58 and AtMYB63 activated lignin biosynthetic genes except *F5H1* [45, 57]. This result is consistent with the observation that the AC cis-acting element, a binding site for AtMYB58, is absent in the promoter of *F5H1* [46, 57]. In *Arabidopsis*, AtSND1 has been reported to regulate *F5H1* gene expression [46]. In the present study, we showed that the expression of *F5H1* is up regulated by PdWND3A overexpression (Fig. 4b). Because AtVNDs (AtVND1 to AtVND7) have been shown to bind to the consensus DNA sequence of secondary wall NAC binding element (SNBE) in the promoter region of a group of genes associated with cell wall biosynthesis [6, 23, 48] and the SNBE consensus is present in the *F5H1* promoter [50], it is plausible that *F5H1* may serve as a direct target PdWND3A. The biochemical determination of *F5H1* as a potential PdWND3A target gene (i.e., via protein-DNA binding assays) deserves further investigation.

### PdWND3A and sugar release

Previous studies using *P. trichocarpa* natural variants showed that both lignin content and S/G ratio affect saccharification efficiency [34, 43]. Glucose release was significantly correlated with both lignin content and S/G ratio [34, 43]. However, the glucose release depended on lignin content but not on S/G ratio when sugar release was measured without pretreatment [34]. In the present study, *OXPdWND3A* transgenic lines showed both higher lignin content and higher S/G ratio (Fig. 3, Fig. 4, Table 1) with lower saccharification efficiency measured without pretreatment (Fig. 5), which is consistent with the observation in *P. trichocarpa* natural variants [34]. Therefore, lignin content seems to play a more dominant role than S/G ratio in the process of saccharification without pretreatment. Collectively, PdWND3A, when overexpressed, negatively impacts saccharification efficiency. As a future study, creating and characterizing *Populus PdWND3A* knockdown or knockout transgenic plants may complement and potentially strengthen the conclusion on the role of PdWND3A in lignin biosynthesis and sugar release drawn from overexpression study.

## Conclusions

Our results indicate that PdWND3A, a member of NAC domain-containing protein family, impacts both lignin biosynthesis and lignin monomer composition. Specifically, PdWND3A regulates the expression of *F5H* gene. Overexpression of PdWND3A negatively impacts saccharification efficiency and biomass production.

## Methods

### Plant materials

The full-length open reading frame of *PdWND3A* was amplified from *Populus deltoides* genotype WV94 and cloned into the pAGW560 binary vector for transformation into WV94. We followed the same procedure for growing and maintaining transgenic plants in the greenhouses as reported in a previous publication [38]. The growth conditions were set with constant 25 °C with 16 h/8 h photoperiod.

### Amino acid sequence alignment and phylogenetic analysis

AtSND1 (AT1G32770) was subjected to Phytozome v12.0 (https://phytozome.jgi.doe.gov) [8] and BLAST (https://blast.ncbi.nlm.nih.gov/Blast.cgi) [1] to identify NAC domain-containing proteins in the *Populus* (*P. trichocarpa*) and *Arabidopsis* (*A. thaliana*) genomes. The full-length amino acid sequence homologs of AtSND1 from each species were subsequently used to perform reciprocal sequence homolog search with > 30% amino acid similarity cutoff (e-value*<* 0.01). The collected proteins were used as subjects in the Pfam database to predict putative protein domains and functional motifs [7]. The phylogenetic tree was constructed by PhyML (a phylogeny software based on the maximum-likelihood principle) using Jones-Taylor-Thornton (JTT) model matrix of amino acid substitution with 1000 bootstrap replication [9]. Nearest-Neighbor-Interchange (NNI) algorithm was used to perform tree topology search.

### Phloroglucinol-HCl staining

To obtain the image of xylem vessel formation from *OXPdWND3A* transgenic plants and WV94 wild-type plants, stem tissues were collected at a position 15 cm above the stem base of 6-month-old plants. Cross-section specimen were sliced at 100 μm thickness without any fixation by using Leica RM2255 microtome (Leica biosystems, IL). Each slice was directly stained in 2% Phloroglucinol (Sigma-Aldrich, St. Louis, MO) dissolved in 95% ethanol for 5 min in dark. The red color was developed by adding 2–3 drops of concentrated Hydrochloride (HCl). Images were captured using SteREO Discovery V8 dissecting microscope (ZEISS, Thornwood, NY). The total count of vessel in each image was determined by ImageJ1 open source program [30].

### RNA extraction and RT-PCR

To measure relative transcript abundance of *PdWND3A* and secondary cell wall biosynthesis-related genes, total RNA was extracted from young stem tissue (1–3 internode) and mature leaf (4-6th from apex) of six-month-old *Populus* plants with Plant Spectrum RNA extraction kit with treatment of in-column DNase following manufacture’s manual (Sigma-Aldrich). We performed quantitative reverse transcription polymerase chain reaction (sq- or qRT-PCR) to determine relative transcript abundance of selected genes. The single strand complementary DNA (cDNA) was synthesized from 1 μg of total RNA by 1 h incubation with RevertAid reverse transcriptase (Thermo Fisher Scientific, Hudson, NH) at 42 °C. One μl of two-times diluted cDNA was used for real time PCR reaction. PCR reaction was performed with Maxima SYBR Green/ROX qPCR master mix including uracyl DNA glycosylase (UDG) (Thermo Fisher Scientific). Gene-specific primers used for PCR reactions were listed in the Additional file 1. PCR reaction was started with UDG activation at 50 °C for 2 min, a pre-denaturation of 95 °C for 10 min, followed by 40 cycles of combined two steps of 95 °C for 15 s and 60 °C for 30 s. The relative gene expression was calculated by 2^–ΔΔ*Ct*^ equation [17]. *Populus UBIQUITIN C* (PdUBCc, Potri.006G205700) was used as an internal control for all relative quantification analyses.

### Chemical composition analysis

Chemical composition, including carbohydrates and lignin of the *OXPdWND3A* transgenic lines, was analyzed and compared with the control (wild-type WV94) by two-step sulfuric acid hydrolysis according to the NREL procedure [32]. Wiley-milled, 6-month-old *Populus* stems were Soxhlet-extracted using ethanol/toluene (1:2, v/v) for 12 h. For the analysis of leaf tissues, additional 12 h ethanol/toluene extraction and 12 h acetone extraction were conducted. The extractives-free samples were air-dried and hydrolyzed by two-step acid method. Briefly, the biomass was hydrolyzed with 72% H_2_SO_4_ at 30 °C for 1 h and 4% H_2_SO_4_ at 121 °C for 1 h. The solid residues were filtered and washed with excessive amounts of deionized water and oven-dried at 105 °C for 24 h. Ash content was measured by muffle furnace at 575 °C for 12 h. Klason lignin content was calculated as below:

$$ Klason\ lignin\ content\ \left[\%\right]=\frac{Acid\ insoluble\ residues\ \left[g\right]- Ash\ \left[g\right]}{Extractives\ free\ sample\ \left[g\right]}\times 100 $$

Carbohydrate contents were analyzed using a Dionex ICS-3000 ion chromatography system with external standards.

### Lignin S/G ratio analysis

Nuclear magnetic resonance (NMR) analysis was used to measure the lignin S/G ratio. Stem samples were extracted as described above. Cellulolytic enzyme lignin was isolated from the extractives-free biomass as described in a previous study [42]. The isolated lignin (~ 30 mg) was dissolved with DMSO-*d*_6_ in 5 mm NMR tube. A Bruker Avance III 400 MHz spectroscopy equipped with a 5 mm Broadband Observe probe and Bruker standard pulse sequence (‘hsqcetgpsi2’) was used for two-dimensional (2D) ^1^H-^13^C heteronuclear single quantum coherence (HSQC) NMR analysis at 300 K. The spectral widths of 11 ppm (^1^H, 2048 data points) and 190 ppm in F1 (^13^C, 256 data points) were employed for the ^1^H and ^13^C-dimensions, respectively. The number of transients was 64 and the coupling constant (^1^*J*_CH_) used was 145 Hz. Bruker Topspin software (v3.5) was used for data processing.

### Saccharification efficiency assay

Stem tissues collected at a position 15 cm above the stem base of 6-month-old plants were dried and Wiley-milled to 40-mesh for sugar release measurement. Approximately 250 mg of sample was placed in 50 mM citrate buffer solution (pH 4.8) with 70 mg/g-biomass of Novozymes CTec2 (Novozymes, Franklinton, NC) loading. The enzymatic hydrolysis was conducted at 50 °C with 200 rpm in an incubator shaker for 48 h. Enzymes in the hydrolysate were deactivated in the boiling water for 5 min prior to the analysis of released sugars by using the Dionex ICS-3000 ion chromatography system. Each analysis was conducted in duplicates from single plant of each transgenic line.

### Statistical analysis

T-test (against WV94) was performed at *p* < 0.01 by t-test function integrated in the Excel software (Microsoft, Redmond, WA) for all statistical analysis. Asterisk in each figure indicates significant difference from WV94 or control samples (*p* < 0.01).

## Supplementary information

**Additional file 1.** The list of primers used for PCR and RT-PCR analyses in this study.

**Additional file 2 **The phylogenetic analysis of *Populus* and *Arabidopsis* VND/NST/SND proteins. (a) PhyML phylogenetic tree analysis. A total of 21 and 22 different loci from *Populus* and *Arabidopsis*, respectively, were identified as AtSND1 homologous proteins. The full-length amino acid sequences were subjected to PhyML. Potri005G018000, a receptor like protein, was used as an outgroup protein sequence in this phylogenic tree. The AtVND homolog cluster is highlighted by green color and these proteins are used for further analyses shown in Fig. 1. (b) Heatmap illustrating amino acid sequence similarity of 43 VND homologs from *Populus* and *Arabidopsis*.

**Additional file 3. **VND homologs in *Populus* and *Arabidopsis*. (a) Phylogenetic tree of *Populus* NAC domain-containing proteins and *Arabidopsis* NAC proteins known as the master switch transcription factors regulating secondary cell wall biosynthesis. Potri.015G127400 (PtrWND3A) shows high amino acid sequence similarity with AtVND4 and VND5. (b) The heatmap illustrating full-length amino acid sequence similarity between VND homologs in *Populus* and *Arabidopsis*. (c) Conserved domain in full-length amino acid sequence of proteins shown in panel (a) and (b). Note that NAC domain is the only conserved region among VND proteins in *Populus* and *Arabidopsis*.

**Additional file 4. **The cDNA sequence alignment between Potri.012G126500 (PdWND3B) and Potri.015G127400 (PdWND3A). The missing sequence region between these two genes is highlighted with yellow color.

**Additional file 5. **Expression of *PdWND3A* in the *Populus* transgenic plants**.** Asterisk marks selected lines for further analysis presented in this study.

## Data Availability

All data generated or analyzed during this study are included in this published article and its supplementary information files.

## Abbreviations

4CL: 4-coumarate:CoA ligase
C4H: Cinnamate 4-hydroxylase
CAD: Cinnamoyl alcohol dehydrogenase
CCoAOMT: Caffeoyl-CoA O-methyltransferase
CCR: Cinnamoyl CoA reductase
F5H: Ferulate 5-hydroxylase
F5H1: FERULATE 5-hydroxylase1
G: Guaiacyl monolignol
H: Hydroxyphenyl monolignol
MYB: V-myb myeloblastosis viral oncogene homolog
NAC: No Apical Meristem (NAM), *Arabidopsis* Transcriptional Activation Factor (ATAF1/2), Cup-shaped Cotyledon (CUC2)
NST1: NAC secondary wall thickening promoting factor1
*OXPdWND3A*: *Populus deltoides* transgenic plants overexpressing *PdWND3A*
PAL: L-phenylalanine ammonia-lyase
PdWND3A: *Populus deltoides* wood-associated NAC domain protein 3A; Potri.015G127400
PdWND3B: *Populus deltoides* wood-associated NAC domain protein 3B; Potri.012G126500
S: Syringyl monolignol
SNBE: Secondary wall NAC binding element
SND1: Secondary wall-associated NAC domanin protein1
TERE: Tracheary-element-regulating *cis*-elements
VND: Vascular-related NAC domain
