# Supplementary material for: PdWND3A, a wood-associated NAC domain-containing protein, affects lignin biosynthesis and composition in Populus
Source: BMC Plant Biol. 2019 Nov 11;19:486. doi: 10.1186/s12870-019-2111-5 (PMC6849256; doi:10.1186/s12870-019-2111-5)
Supplement: Supplementary file 2 — Additional file 2 The phylogenetic analysis of Populus and Arabidopsis VND/NST/SND proteins. (a) PhyML phylogenetic tree analysis. A total of 21 and 22 different loci from Populus and Arabidopsis, respectively, were identified as AtSND1 homologous proteins. The full-length amino acid sequences were subjected to PhyML. Potri005G018000, a receptor like protein, was used as an outgroup protein sequence in this phylogenic tree. The AtVND homolog cluster is highlighted by green color and these proteins are used for further analyses shown in Fig. 1. (b) Heatmap illustrating amino acid sequence similarity of 43 VND homologs from Populus and Arabidopsis. [file 12870_2019_2111_MOESM2_ESM.docx]

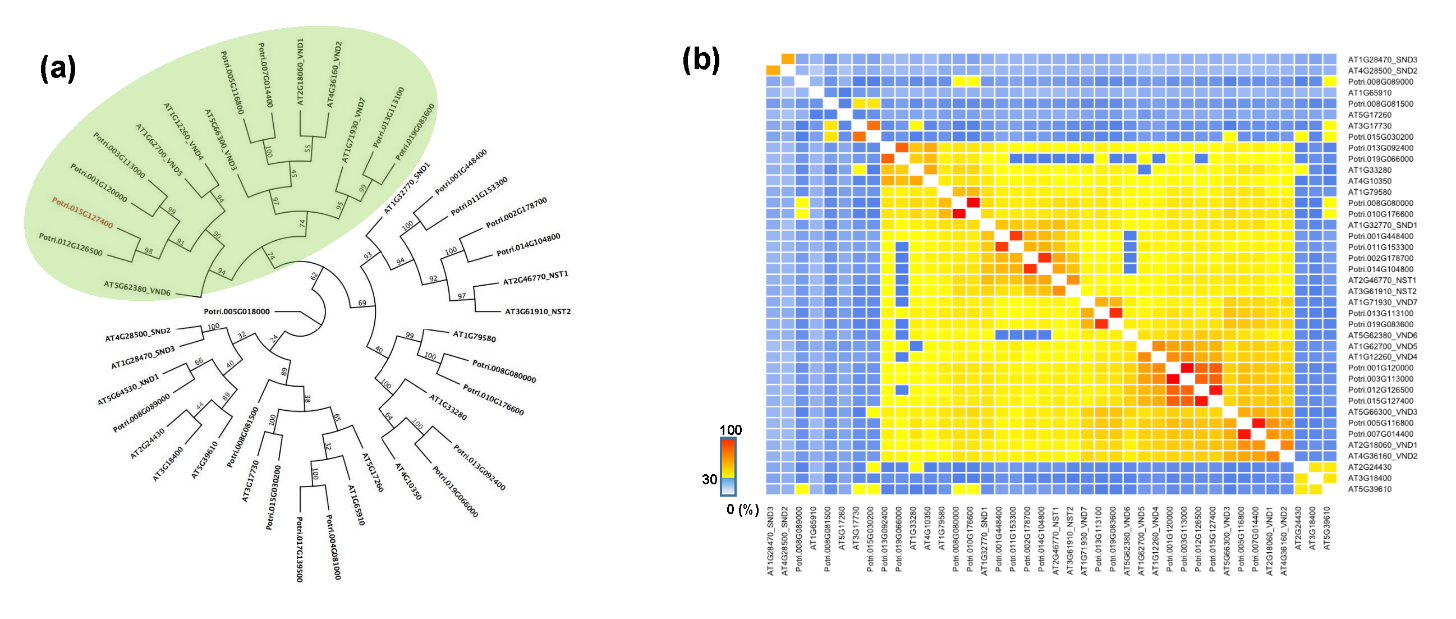


**Additional file 2. The phylogenetic analysis of *Populus* and *Arabidopsis* VND/NST/SND proteins.** (a) PhyML phylogenetic tree analysis. A total of 21 and 22 different loci from *Populus* and *Arabidopsis*, respectively, were identified as AtSND1 homologous proteins. The full-length amino acid sequences were subjected to PhyML. Potri005G018000, a receptor like protein, was used as an outgroup protein sequence in this phylogenic tree. The AtVND homolog cluster is highlighted by green color and these proteins are used for further analyses shown in Figure 1. (b) Heatmap illustrating amino acid sequence similarity of 43 VND homologs from *Populus* and *Arabidopsis*.
