# Supplementary figures and images for: PdWND3A, a wood-associated NAC domain-containing protein, affects lignin biosynthesis and composition in Populus
Source: BMC Plant Biol. 2019 Nov 11;19:486. doi: 10.1186/s12870-019-2111-5 (PMC6849256; doi:10.1186/s12870-019-2111-5)

## Slide 1
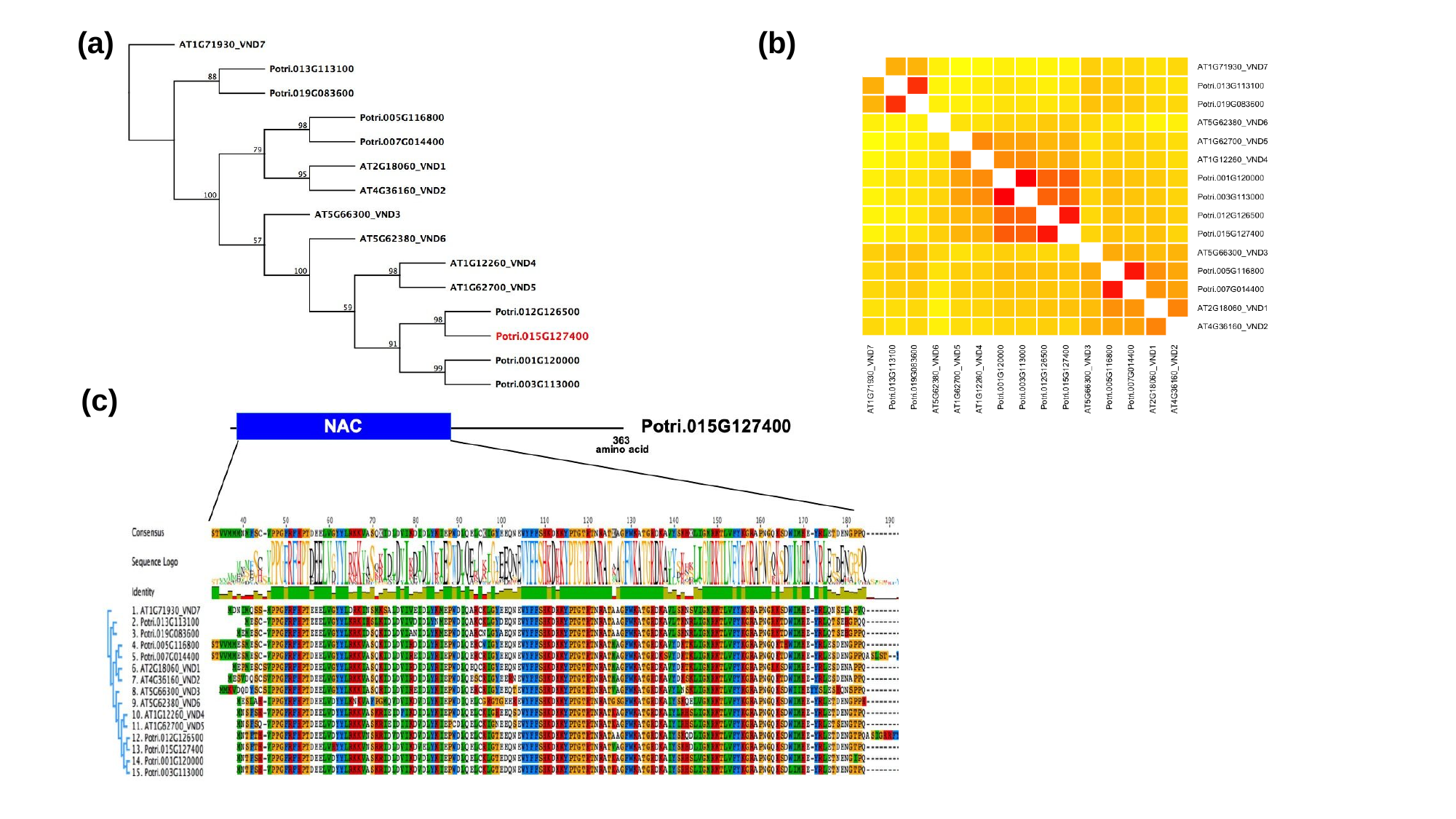

(a)
(b)
(c)

Supplement: Supplementary file 3 — Additional file 3. VND homologs in Populus and Arabidopsis. (a) Phylogenetic tree of Populus NAC domain-containing proteins and Arabidopsis NAC proteins known as the master switch transcription factors regulating secondary cell wall biosynthesis. Potri.015G127400 (PtrWND3A) shows high amino acid sequence similarity with AtVND4 and VND5. (b) The heatmap illustrating full-length amino acid sequence similarity between VND homologs in Populus and Arabidopsis. (c) Conserved domain in full-length amino acid sequence of proteins shown in panel (a) and (b). Note that NAC domain is the only conserved region among VND proteins in Populus and Arabidopsis. [file 12870_2019_2111_MOESM3_ESM.pptx]
