# Supplementary material for: PdWND3A, a wood-associated NAC domain-containing protein, affects lignin biosynthesis and composition in Populus
Source: BMC Plant Biol. 2019 Nov 11;19:486. doi: 10.1186/s12870-019-2111-5 (PMC6849256; doi:10.1186/s12870-019-2111-5)
Supplement: Supplementary file 4 — Additional file 4. The cDNA sequence alignment between Potri.012G126500 (PdWND3B) and Potri.015G127400 (PdWND3A). The missing sequence region between these two genes is highlighted with yellow color. [file 12870_2019_2111_MOESM4_ESM.docx]

**Additional file 4. The cDNA sequence alignment between Potri.012G126500 (PdWND3B) and Potri.015G127400 (PdWND3A).**

The missing sequence region between these two genes is highlighted with yellow color.

Score = 808.5, Identities = 1005/1116 (90%), 
Positives = 1005/1116 (90%), Gaps = 27/1116 (2%)

Potri.012G126500      1 ATGAATACTTTTACACATGTTCCTCCTGGTTTCCGGTTCCATCCTACCGACGAAGAACTT   60 
                        ATGAAT CTTTTACACA GTTCCTCCTGGTTT CG TTCCATCCTACCGA GAAGAACTT      
Potri.015G127400      1 ATGAATTCTTTTACACACGTTCCTCCTGGTTTTCGATTCCATCCTACCGATGAAGAACTT   60 

Potri.012G126500     61 GTCGATTACTACCTTAGGAAAAAAGTGAATTCAAGAAGGATTGATGTAGATGTCATTAAA  120 
                        GTC ATTACTACCTTAG AAAAAAGT AATTCAAGAAGGATTGA  TAGATGTCATTAA       
Potri.015G127400     61 GTCCATTACTACCTTAGAAAAAAAGTAAATTCAAGAAGGATTGACCTAGATGTCATTAAG  120 

Potri.012G126500    121 GATGTCGACCTTTACAAAATTGAGCCATGGGATCTTCAAGAACTATGCCGAATAGGAACC  180 
                        GATGTCGA CTTTACAA ATTGAGCCATGGGATCTTCAAGAACT TGCCGAATAGGAACC      
Potri.015G127400    121 GATGTCGAGCTTTACAAGATTGAGCCATGGGATCTTCAAGAACTGTGCCGAATAGGAACC  180 

Potri.012G126500    181 GAGGAACAAAATGAATGGTACTTTTTTAGCCACAAAGATAAGAAGTATCCAACTGGAACT  240 
                        GAGGA CAAAATGAATGGTACTTTTTTAGCCACAAAGATAAGAAGTATCC ACTGG ACT      
Potri.015G127400    181 GAGGAGCAAAATGAATGGTACTTTTTTAGCCACAAAGATAAGAAGTATCCTACTGGGACT  240 

Potri.012G126500    241 CGCACAAATAGAGCCACTGCTGCTGGGTTTTGGAAAGCAACAGGTAGAGACAAGGCAATT  300 
                        CGCACAAATAGAGCCACTG TGC GG TTTTGGAAAGCAACAGGTAGAGACAAGGCAATT      
Potri.015G127400    241 CGCACAAATAGAGCCACTGTTGCAGGATTTTGGAAAGCAACAGGTAGAGACAAGGCAATT  300 

Potri.012G126500    301 TATTCGAAGCAAGACTTGATCGGAATGAGGAAGACCTTAGTCTTTTATAAAGGTCGAGCT  360 
                        TATTCGAAGCA GACTTGATCGGAATGAGGAAGACCTTAGTCTTTTATAAAGGTCGAGCT      
Potri.015G127400    301 TATTCGAAGCACGACTTGATCGGAATGAGGAAGACCTTAGTCTTTTATAAAGGTCGAGCT  360 

Potri.012G126500    361 CCGAATGGGCAGAAATCGGACTGGATTATGCATGAATACCGACTCGAAACAGATGAAAAT  420 
                        CCGAATGG CAGAAATCGGACTGGATTATGCATGA TAC GACT GAAACAGATGAAAAT      
Potri.015G127400    361 CCGAATGGACAGAAATCGGACTGGATTATGCATGAGTACAGACTTGAAACAGATGAAAAT  420 

Potri.012G126500    421 GGGACTCCACAGGCAAGTATAGGAAGACGTTTTTATGAAGAAGGTTGGGTCGTGTGTAGG  480 
                        GGGACTCCACAGG                        AAGAAGGTTGGGT GTGTG AGG      
Potri.015G127400    421 GGGACTCCACAGG------------------------AAGAAGGTTGGGTTGTGTGCAGG  456 

Potri.012G126500    481 GTGTTCAAGAAAAGATTACCAACGATGCGAAAAGTGAGTGAGCATGAATCAGTCTGTTGG  540 
                        GTGTTCAAGAA AGA TACCAACGATGC AAAAGTGAGTGAGCATGAATCAGT TG TGG      
Potri.015G127400    457 GTGTTCAAGAAGAGAATACCAACGATGCCAAAAGTGAGTGAGCATGAATCAGTTTGCTGG  516 

Potri.012G126500    541 TATGATGATCAAGTTGCATTCATGCATGACTTGGATTCACCAAAGCAAAATTCTCAGCCT  600 
                        TATGATGATCAAGTTGCATTCATGCATGACTTGGATTCACCAAAGCAAAA T  CAGCCT      
Potri.015G127400    517 TATGATGATCAAGTTGCATTCATGCATGACTTGGATTCACCAAAGCAAAACTTCCAGCCT  576 

Potri.012G126500    601 GATTTGGGTTATCAATTCCCATACCCCTGCAAGAAAGAGATAGATCTGCAGTACCAAATC  660 
                        GA TT GGTTATCAATTCCC TAC CCTGCAAGAAAGAG T GAT T CAGTACCAAATC      
Potri.015G127400    577 GACTTAGGTTATCAATTCCCTTACTCCTGCAAGAAAGAGCTGGATTTTCAGTACCAAATC  636 

Potri.012G126500    661 CCTCATGATCACTTCCTCCAACTCCCACTTCTACAAAGCCCTAAACTGCTGCAACCAGCT  720 
                        CCTCATGATCACTTCCTCCA CTCCCACTTCTACAAAG CCTAAACT CTGCAA C GCT      
Potri.015G127400    637 CCTCATGATCACTTCCTCCAGCTCCCACTTCTACAAAGTCCTAAACTTCTGCAATCGGCT  696 

Potri.012G126500    721 CCAACTATAAGCTGCAATTCCATCAATGCTGCATATGGCCTGGACATAAACCAGGCAAGC  780 
                        CC AC  TAAG TGCAATTCCATCAATGCTGCATATGGCCT GACATAAACCAG CAAGC      
Potri.015G127400    697 CCTACATTAAGTTGCAATTCCATCAATGCTGCATATGGCCTAGACATAAACCAGACAAGC  756 

Potri.012G126500    781 ACTATACAATCCTCAACACTCACACAAGAAGATCACATTCAACAAGCACATGAGCAAAGC  840 
                        ACT T CAATCCTCAACACTCACACAAGAAGATCAC TTCAACAA CACATGAGCAAAG       
Potri.015G127400    757 ACTTTTCAATCCTCAACACTCACACAAGAAGATCACGTTCAACAAACACATGAGCAAAGA  816 

Potri.012G126500    841 TTTCCCTCTATCTATGGTAGCAACAACATTAAT---GAGCAAGCAGTTGATCAAGTGACA  897 
                        TTTCCC C ATCTATGGTA CAACAACA  AAT   GAGCAAGCAGTTGATCAAGT ACA      
Potri.015G127400    817 TTTCCCACCATCTATGGTAACAACAACAGCAATCATGAGCAAGCAGTTGATCAAGTCACA  876 

Potri.012G126500    898 GACTGGAGAGTACTTGACAAATTTGTTGCTTCTCAATTGAGCCAAGAAGATGTGGTCAAG  957 
                        GACTGGAGAGT CTTGACAAATTTGTTGCTTCTCAATTGAGCCA GAAGATGTG TCAAG      
Potri.015G127400    877 GACTGGAGAGTGCTTGACAAATTTGTTGCTTCTCAATTGAGCCACGAAGATGTGATCAAG  936 

Potri.012G126500    958 GAAAACAACCACCCAAATGCCAGCAACAACATCTTCAACACATCATCAATCCATGCACCA 1017 
                        GAAA  AACCACCCAAATGC AGCAACAACAT TTCAACACATCATCA TCCA GCA CA      
Potri.015G127400    937 GAAACTAACCACCCAAATGCTAGCAACAACATTTTCAACACATCATCAGTCCACGCAGCA  996 

Potri.012G126500   1018 AACATATTTGCTCGACACTTGAGTAAGCAAGATACATCCGTGCCGGAAAATGCCTCGACG 1077 
                        AACATA TTG T GACACTTGA TAAGCAAGA  CATCCGTGC GGAAAA GCCTCGACG      
Potri.015G127400    997 AACATACTTGTTAGACACTTGAATAAGCAAGAAGCATCCGTGCAGGAAAACGCCTCGACG 1056 

Potri.012G126500   1078 TCAACCTCCAGTTGTCAAATTGATCTATGGAAATGA 1113 
                        TCAACCTCCAGTTGTCAAATTGATCTATGGAA TGA      
Potri.015G127400   1057 TCAACCTCCAGTTGTCAAATTGATCTATGGAAGTGA 1092
