# Supplementary material for: PdWND3A, a wood-associated NAC domain-containing protein, affects lignin biosynthesis and composition in Populus
Source: BMC Plant Biol. 2019 Nov 11;19:486. doi: 10.1186/s12870-019-2111-5 (PMC6849256; doi:10.1186/s12870-019-2111-5)
Supplement: Supplementary file 5 — Additional file 5. Expression of PdWND3A in the Populus transgenic plants. Asterisk marks selected lines for further analysis presented in this study. [file 12870_2019_2111_MOESM5_ESM.docx]

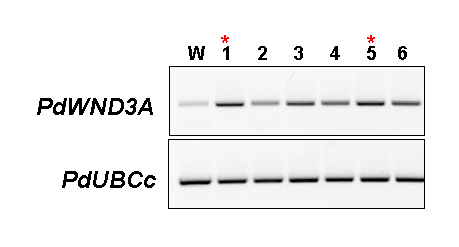


**Additional file 5. Expression of *PdWND3A* in the *Populus* transgenic plants.** Asterisk marks selected lines for further analysis presented in this study.
